# Multimodal diagnosis of Alzheimer’s disease based on resting-state electroencephalography and structural magnetic resonance imaging

**DOI:** 10.3389/fphys.2025.1515881

**Published:** 2025-03-12

**Authors:** Junxiu Liu, Shangxiao Wu, Qiang Fu, Xiwen Luo, Yuling Luo, Sheng Qin, Yiting Huang, Zhaohui Chen

**Affiliations:** ^1^ Guangxi Key Laboratory of Brain-inspired Computing and Intelligent Chips, School of Electronic and Information Engineering, Guangxi Normal University, Guilin, China; ^2^ Key Laboratory of Nonlinear Circuits and Optical Communications, Education Department of Guangxi Zhuang Autonomous Region, Guangxi Normal University, Guilin, Guangxi, China; ^3^ Xiangsihu College, Guangxi University for Nationalities, Nanning, China

**Keywords:** Alzheimer’s disease, electroencephalography, magnetic resonance imaging, multimodal, joint attention mechanism

## Abstract

Multimodal diagnostic methods for Alzheimer’s disease (AD) have demonstrated remarkable performance. However, the inclusion of electroencephalography (EEG) in such multimodal studies has been relatively limited. Moreover, most multimodal studies on AD use convolutional neural networks (CNNs) to extract features from different modalities and perform fusion classification. Regrettably, this approach often lacks collaboration and fails to effectively enhance the representation ability of features. To address this issue and explore the collaborative relationship among multimodal EEG, this paper proposes a multimodal AD diagnosis model based on resting-state EEG and structural magnetic resonance imaging (sMRI). Specifically, this work designs corresponding feature extraction models for EEG and sMRI modalities to enhance the capability of extracting modality-specific features. Additionally, a multimodal joint attention mechanism (MJA) is developed to address the issue of independent modalities. The MJA promotes cooperation and collaboration between the two modalities, thereby enhancing the representation ability of multimodal fusion. Furthermore, a random forest classifier is introduced to enhance the classification ability. The diagnostic accuracy of the proposed model can achieve 94.7%, marking a noteworthy accomplishment. This research stands as the inaugural exploration into the amalgamation of deep learning and EEG multimodality for AD diagnosis. Concurrently, this work strives to bolster the use of EEG in multimodal AD research, thereby positioning itself as a hopeful prospect for future advancements in AD diagnosis.

## 1 Introduction

AD is a neurodegenerative disease with a high incidence rate, currently affecting about 51.6 million people worldwide Schlachetzki et al. (2013), which brings a heavy burden to society. According to reports, 6.7 million Americans aged 65 and older are currently living with Alzheimer’s dementia. This number is likely to grow to 13.8 million by 2060. Meanwhile, the total cost of healthcare, long-term care, and hospice services for people with dementia aged 65 and over will reach an estimated $ 345 billion in 2023 Saykin et al. (2010). So far, many markers of AD have been discovered. Many studies have focused on various aspects such as biomarker discovery, diagnosis methods, and therapeutic strategies. For example, studies with the Zong et al. (2024) proposed innovative approaches in analyzing neuroimaging data for AD diagnosis by integrating advanced image processing algorithms and machine learning techniques, aiming to improve the accuracy and efficiency of diagnosis. Another study with Zuo et al. (2024) focused on using multimodal data fusion methods to extract more comprehensive features from different sources of AD-related data for better understanding the disease progression. Moreover, the research with Pan et al. (2024) explored the potential of using specific neural network architectures to enhance the performance of AD diagnosis based on neuroimaging data. And the work with Zuo et al. (2023) investigated how to utilize time-series information in different modalities to capture the dynamic changes of AD, which is also quite inspiring for the field. The Alzheimer’s Disease Neuroimaging Initiative (ADNI) has played a significant role in biomarker research, serving as a milestone in the field. Its primary objective is to the development of AD research by collecting various candidate biomarkers. ADNI combines magnetic resonance imaging (MRI) Elshafey et al. (2014) and positron emission tomography (PET) scans to study AD. It encompasses a vast amount of information related to the genetics, cerebrospinal fluid, and other biomarkers associated with AD a. Illán et al. (2011). But these modalities lack temporal resolution, and their analysis is only focused on traditional visual inspection. In recent years, some studies related to AD have been exploring the use of electroencephalography (EEG) to detect AD Darves-Bornoz et al. (2023). At the same time, studies have also shown that EEG patterns are also one of the biomarkers of AD. In recent years, there has been a growing interest in AD diagnosis research using medical neuroimaging. Both machine learning (Peng et al., 2022; Uysal and Ozturk, 2020; Franciotti et al., 2023) and deep learning methods Alorf and Khan (2022); Leela et al. (2023); EL-Geneedy et al. (2022); Yao et al. (2023a) have been widely explored in this field. However, medical neuroimaging lacks time resolution in the resting state, and it is difficult to form a continuous onset period time. To address this issue, researchers have turned to EEG as a potential marker for AD diagnosis, as EEG patterns provide temporal resolution. However, extracting meaningful representations from EEG patterns remains a significant challenge. Fortunately, deep learning models have also been applied to automatic feature extraction of EEG modalities Bi and Wang (2019); Xia et al. (2023), which can reduce the problems caused by the feature extraction process. The AD EEG data of the corresponding channel is selected, the corresponding channel data features are extracted and learned and finally classified by deep learning or machine learning classifier. Both medical neuroimaging and EEG data possess distinct modal characteristics. Medical neuroimaging captures changes in blood oxygen levels and changes in the hippocampus, while EEG provides high temporal resolution information. Integrating these two modes has been a challenging task for researchers. In recent years, there has been rapid development in multimodal AD diagnosis (Velazquez and Lee, 2022; Eslami et al., 2023; Chen et al., 2023; Leng et al., 2023), with most studies focusing on combining medical neuroimaging with clinical data. However, most multimodal models are trained independently for each modality, failing to capture the correlation and dependence between modalities. Only a few studies Jesus et al. (2021); Colloby et al. (2016) have explored multimodal approaches using EEG data, employing machine learning methods for AD prediction. However, these studies rely on manual feature extraction, which is time-consuming, lacks interactivity, and is subject to subjective bias. Furthermore, identify and incorporate hidden features between the data. This study addresses the following issues. First, the absence of automatic feature extraction capabilities, compounded by the subjective nature of feature extraction, poses significant hurdles in identifying and extracting latent features from the data. Second, the current practice of training each modality model independently overlooks the interdependence and correlation between modalities. To overcome these obstacles, our work proposes a novel multimodal model that integrates sMRI and EEG patterns. Leveraging the unique characteristics of sMRI and EEG modalities, sMRI-based convolutional neural network (sCNN-sMRI) and EEG-based convolutional neural network (sCNN-EEG) are designed to extract features from respective modal data and promote the interdependence and correlation between different features. Furthermore, this work introduces an attention mechanism to bridge the semantic gap between models, thereby enhancing the learning capacity of the overall model. The main contributions of this paper are as follows. a. A multimodal AD diagnosis model based on the convolutional neural network (MCNNRF) is proposed. b. This work devises dedicated network architectures, namely sCNN-EEG and sCNN-sMRI, tailored for processing EEG and sMRI data, respectively. c. To handle the complexity of feature mapping and unveil latent features, stacked Random Forests (RF) is used for classification tasks d. A groundbreaking multimodal joint attention mechanism (MJA) is introduced to address the intricacies of feature extraction across different modalities. This mechanism fosters synergistic feature extraction while facilitating collaboration between modalities, thereby enhancing the model’s ability to represent features effectively.

The rest of this work is organized as follows. The related work is discussed in Section II. The model used and constructed are provided in Section III. The model evaluation and experiments are presented in Section IV, and Section V concludes this paper.

## 2 Related Works

Currently, there are several studies focusing on AD diagnosis using unimodal data, primarily focusing on medical neuroimaging techniques. In Liu et al. (2023), a diagnostic method using two - sample t - tests to detect AD is proposed. First, it uses two - sample t - tests to detect AD - related regions in MRI, then extracts the features of related regions through an unsupervised learning neural network, and finally classifies AD using a clustering algorithm. In Mehmood et al. (2021), a layer - by - layer transfer learning model for AD diagnosis is developed.

However, the above-mentioned studies are all unimodal studies, lacking the interaction between modes and not considering the complementarity between multi-modalities. Multimodality has gained significant popularity in recent years, and a plethora of studies explore the potential of combining multiple modalities to enhance analysis and understanding. In Colloby et al. (2016), multimodal EEG - MRI in the differential diagnosis of AD and dementia with Lewy bodies is proposed. The MRI index in this work is derived from the medial temporal lobe atrophy (MTA) score. Logistic regression analysis identified EEG predictors for AD and DLB. A joint EEG - MRI model is then generated to examine whether there is an improvement in classification compared to the individual patterns. In Jesus et al. (2021), a multimodal prediction of Alzheimer’s disease severity based on resting - state EEG and structural MRI is proposed. This work investigates the multimodal prediction of Mini - Mental State Examination (MMSE) scores using resting - state electroencephalography (EEG) and structural magnetic resonance imaging (MRI) scans. Evaluation is performed by three feature selection algorithms and four machine learning algorithms. Compared with Colloby et al. (2016), this study is not only focused on the differential diagnosis between AD and other diseases but also aims to build a general multimodal diagnosis model for AD. In terms of methods, Colloby et al. (2016) relies on manually extracted MRI indicators and logistic regression analysis, while this study automatically extracts features from EEG and sMRI through deep learning, improving the accuracy and efficiency of diagnosis. Compared with Jesus et al. (2021), this study innovatively proposes the Multimodal MJA, which effectively promotes the collaboration between different modalities. The MJA is more efficient in feature extraction and fusion, thus improving the diagnostic performance. In summary, previous AD diagnosis studies have achieved certain results in both unimodal and multimodal fields. However, most studies suffer from insufficient collaboration between modalities and less intelligent feature extraction methods. This study addresses these issues by designing dedicated feature - extraction models sCNN - EEG and sCNN - sMRI, combined with the innovative MJA, providing a more effective method for AD diagnosis.

## 3 Methods

In this section, the dataset is described in Section A. The feature selection method is provided in Section B. The sCNN-EEG model is proposed in Section C. The sCNN-sMRI model is proposed in Section D. The MJA module is described in Section E, and finally, the MCNNRF model is proposed in Section F.

### 3.1 Dataset

The data set used in this work was provided by Chen et al. (2023). The acquired data underwent text data processing using the Statistical Package for Social Sciences software (SPSS ver. 22.0, http://www01.ibm.com/software/analytics/spss/products/statistics/). During the data processing process, unknown and null values in MRI and EEG were estimated and filled using the weighted nearest neighbor algorithm Troyanskaya et al. (2001). Subsequently, min-max normalization was applied to all data within the range [0,1].

### 3.2 Feature selection

Given that both modes contain hidden features in addition to observed features, utilizing too many features could lead to significant overfitting problems in the model. Therefore, this work uses two feature selection methods to address this concern. MRMR Zhao et al. (2019) feature selection is used for dimensionality reduction of the MRI and EEG datasets. By examining the score values of different subsets of the data set, the highest and most optimal feature set is selected. To streamline the process, a grading strategy of 10 is used for feature selection. The MRMR algorithm is run for each grading label, and N optimal features are chosen within the range of 10 500 through evaluating the score value. Following the selection of the N best features, a feature importance algorithm is employed to verify and further optimize these selected features.

### 3.3 Model for unimodal EEG data

**FIGURE 1:**
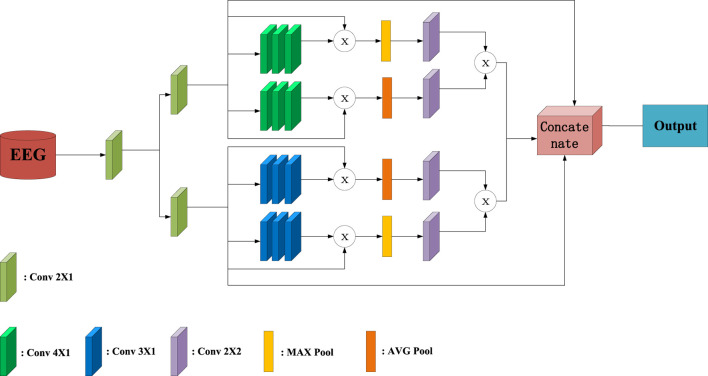
Overall Structure Diagram of sCNN-EEG.

In this work, the sCNN-EEG is designed to extract the important and hidden feature extraction from AD EEG data. In Figure 1, the input undergoes convolution two kernels of size of 2, resulting in the generation of matrix $X_{1}$ . $X_{1}$ is then processed through two different branches, where stacked convolutions with kernel sizes of 3 and 4 are applied, producing $X_{2}$ and $X_{3}$ , respectively. Next, matrix multiplication is performed between $X_{1}$ and $X_{2}$ , yielding a matrix graph $S_{1}$ that contains both important and hidden features. A similar approach is used to combine $X_{1}$ and $X_{3}$ , resulting in the formation of $S_{2}$ . The matrix graph $S_{1}$ can be calculated by Equation 1

$$S_{1}=X_{1}\times X_{2}.$$ (1)

The convolution kernels of the convolutional layer are all initialized with constants. A stride value of 1 is used to move the kernel window and perform the convolution across the entire input matrix. The sCNN-EEG maintains the size of the convolutional feature map, akin to the feature map of the previous convolution, and preserves the shape of the input data by setting the padding variables to be the same. The same padding variable can ensure that there will be no matrix problems during subsequent fusion. Since the second half of the pooling layer has the same structure, take the part of the model where $S_{1}$ is an example. The feature map $S_{1}$ is taken as two inputs, which are processed by the max pooling layer and the average pooling layer, respectively. After processing, two feature maps ( $M_{1}$ and $M_{2}$ ) are obtained. Both $M_{1}$ and $M_{2}$ subjected to an identical convolution process, employing a kernel size of 2, resulting in the creation of two additional feature maps, $M_{3}$ and $M_{4}$ . Then, a matrix multiplication operation is performed on $M_{3}$ and $M_{4}$ to yield the composite feature map $M_{5}$ . By using pooling operations such as max pooling and average pooling, important spatial information from the input feature maps is preserved while reducing dimensionality. These pooled feature maps, $M_{1}$ and $M_{2}$ , are then processed using convolution, which helps in extracting meaningful features. Finally, matrix multiplication is applied to combine these features, capturing the relationships between the different pooled representations and creating a composite feature map. This process is formally represented by Equation 2, which describes the mathematical operation involved in computing $M_{1}$ .

$$M_{1}=AVG\left(S_{1}\right)\times MAX\left(S_{1}\right),$$ (2)

where AVG here is the average pooling layer, and MAX is the maximum pooling layer.

The two deep feature maps are matrix multiplied and concatenated with the original features to form the final feature map. The purpose of this is to ensure the integrity of feature information and to dig out deep features. Finally, the final feature maps are passed to the max pooling and connection layers. At the same time, the stacked feature maps are flattened using a flattening layer before the connection layer. This allows the feature maps to be transformed into a one-dimensional representation. The connection layer consists of 150, 100, and 50 units, which the flattened feature maps are connected to. Additionally, there is a hidden layer with a 50% dropout rate, which helps prevent overfitting by randomly dropping out half of the units during training. To ensure that the features are non-linear, the connection layer uses the hyperbolic tangent function as the activation function and initializes its weights with the Glorot normal initializer Glorot and Bengio (2010). Therefore, the cost function can be obtained by

$$\begin{array}{cc} L\left(x_{t},y_{t}\right) & =\frac{-1}{N}\times \overset{N}{\underset{i=0}{\sum }}\left(x_{t}\left(i\right)\times \log\left(y_{t}-\left(1-x_{t}\left(i\right)\right)\right)\right.\\ & \;\left.\times \;\log\left(1-x_{t}\right)\right)+\frac{1}{2}\times \partial \times \overset{K}{\underset{k=0}{\sum }}W_{k}^{2}, \end{array}$$ (3)

where L is cost function is the combination of the binary cross-entropy and L2 regularization term. ∂ is a hyper-parameter which represents the regularization coefficient. $x_{t}$ is true class label. $y_{t}$ is predicted class label. N is batch size. $W_{k}$ is the k−th weight parameter of the model. K is the number of weight matrices.

### 3.4 Model for unimodal sMRI data

**FIGURE 2:**
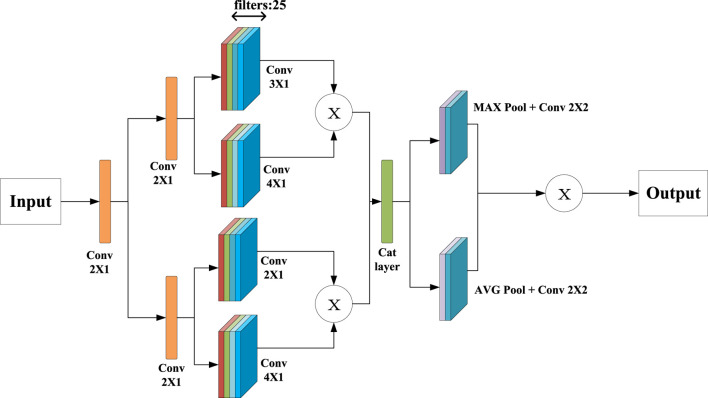
Verall Structure Diagram of sCNN-sMRI.

In this work, the sCNN-sMRI is focused on important feature extraction and hidden feature extraction for sMRI. In Figure 2, the sMRI data is taken as input, first passing through two identical convolution processes, using a convolution kernel of size 2. Then it goes through the feature extraction modules of two different convolution kernels. One of the paths consists of a set of convolution kernels 3 and 4. It aims to create two different feature maps (represented as $a_{1}$ and $a_{2}$ respectively). The other path consists of convolution kernels 4 and 2, which focuses on obtaining two different feature maps (represented as $a_{3}$ and $a_{4}$ respectively). Multiple feature maps containing different feature information are created and fused through different feature extractors. Now all feature map features of the same feature extractor are fused together to form new feature maps $Q_{1}$ and $Q_{2}$ . The $Q_{1}$ and $Q_{2}$ can be obtained by Equations 4, 5

$$Q_{1}=a_{1}\times a_{2},$$ (4)

$$Q_{2}=a_{3}\times a_{4}.$$ (5)

In this model, multiple feature maps obtained from each branch are fused through matrix multiplication. Multiple feature maps $Q_{1}$ and $Q_{2}$ are concatenated to obtain the feature map $A_{1}$ . After cascading, $A_{1}$ is calculated by the maximum pooling and the average pooling respectively, and obtains a multi-pooling feature matrix map. Multi-pooling features are convolved to obtain deep feature maps (each convolution kernel size of 2). The final feature map $A_{2}$ is obtained by matrix multiplication of the maximum feature map and the average feature map. The $A_{1}$ and $A_{2}$ can be obtained by Equations 6, 7

$$A_{1}=Q_{1}\text{©}Q_{2},$$ (6)

$$A_{2}=AVG\left(A_{1}\right)*W\times MA\times \left(A_{1}\right)*W,$$ (7)

where © is the concatenation symbol, AVG is the average pooling layer, and MAX is the maximum pooling layer, W is the convolution kernel size. Finally, the $A_{2}$ is calculated by the max pooling and fully connection layers. After the maximum pooling layer, the structure of the connection layer is consistent with the connection of sCNN-EEG. Both consist of 200, 150, 50% and 50% dropout. The training cost function for this model is the same as Equation 3.

### 3.5 Multimodal joint attention mechanism

**FIGURE 3:**
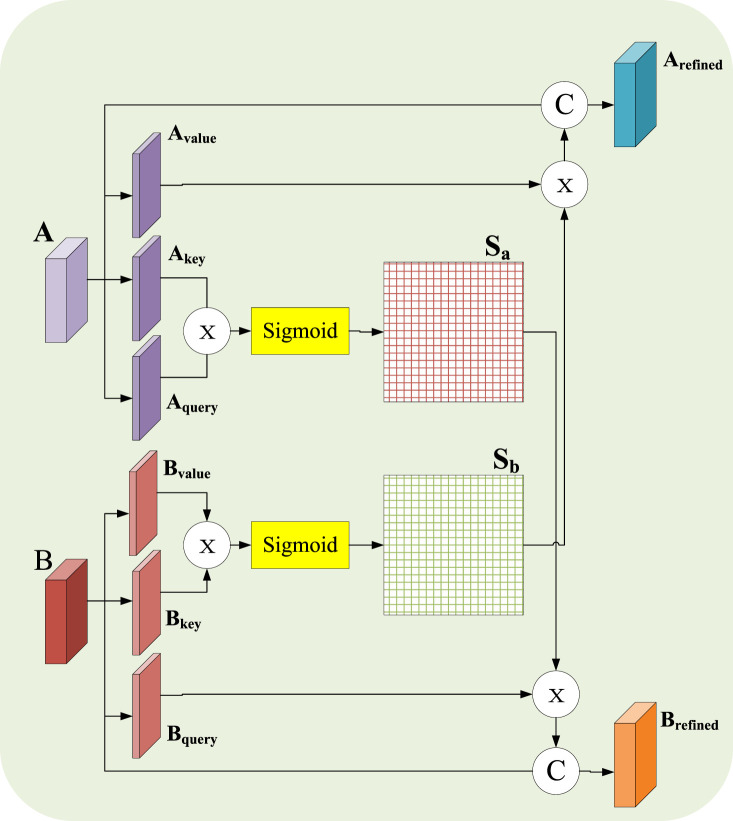
Chematic diagram of MJA module.

During the experiment, it is found that in the multi-branch feature extraction process of MCNNRF, the feature extraction process of the two modalities is independent of each other, and the lack of relevant cooperation may cause the extracted features to be independent of each other. At the same time, it may cause poor representation ability after multimodal fusion. To this end, this work proposes a fusion module of MJA, which is mainly used to explore the deep cooperation of two modalities to enhance the representation ability of extracted features. The structure of the MJA fusion module is shown in Figure 3. The design of the module is inspired by the spatial attention mechanism in Fu et al. (2019). Specifically, the module takes the EEG data branching model (denoted as A) and the sMRI branching model (denoted as B) as input sources. To simplify the description, only the spatial attention unit of branch A is explained in detail in this paper. In the module, the structure of A and B is similar, and each branch consists of three convolutional layers and an S-shaped activation function; the convolutional layers are used to extract the features of each model, and the S-shaped activation function is used for nonlinear transformation. Given the input $N\in R^{\left(G\times H\times W\right)}$ , where G , H , and W denote the number of channels, height, and width of the features, respectively, firstly, $A_{\mathrm{query}}$ , $A_{\mathrm{key}}$ , and $A_{\mathrm{value}}$ are generated by three 1×1 convolution operations, respectively, and the dimensionality of these outputs are all $R^{\left(G/8\times H\times W\right)}$ . In order to reduce the computational cost, the 1×1 convolution reduces the number of channels to 1/8 of the original, thus reducing the computational effort. Next, the attention score matrix is obtained by multiplying the transpose of $A_{\mathrm{query}}$ with $A_{\mathrm{key}}$ . Then, the sigmoid activation function is used to generate the spatial attention graph $S_{a}\in R^{\left(N\times N\right)}$ , which reflects the spatial importance of the input features. Next, for branch B, the same operation is performed to obtain the corresponding spatial attention map $S_{b}\in R^{\left(N\times N\right)}$ , which is used to characterize the spatial feature importance of branch B. Unlike the use of softmax to generate $S_{a}$ in the original spatial attention mechanism, this paper employs an S-shaped activation function, which is designed to capture hidden features over a wider range and to be consistent with the activation function in multimodal models. In addition, in the traditional spatial attention mechanism, $S_{a}$ is only used to refine A. In this design, $S_{b}$ is not only used to refine B, but also achieves a deep fusion of the two modes by combining it with $S_{a}$ , which enables the two modes to work more closely together, thus improving the overall feature representation capability. Therefore, the MJA fusion module is able to better coordinate the feature learning of the two modalities by introducing a joint attention mechanism, which not only weights the features of the respective modalities at the spatial level, but also interactively fuses between the two modalities, thus improving the model’s ability to comprehend and process multimodal data. This design allows the model to extract and fuse information more effectively, enhancing the robustness and accuracy of the final representation.

Specifically, first perform three 1×1 convolutions to generate $A_{\mathrm{query}}$ , $A_{\mathrm{key}}$ and $A_{\mathrm{value}}$ respectively, and make their dimensions controlled at $R^{\left(G\times N\right)}$ Then $A_{\mathrm{value}}$ , $B_{\mathrm{value}}$ , and the corresponding generated $S_{a}$ and $S_{b}$ are matrix multiplied to obtain two attention feature maps $C_{a}$ and $C_{b}$ . The specific formulas of $C_{a}$ and $C_{b}$ can be calculated by Equations 8, 9

$$C_{a}=A_{\mathrm{value}}\times S_{b},$$ (8)

$$C_{b}=B_{\mathrm{value}}\times S_{a}.$$ (9)

In the formula, $C_{a}\epsilon R^{\left(G\times N\right)}$ is the stacked EEG feature guided by the sMRI feature, and $C_{b}\epsilon R^{\left(G\times N\right)}$ is the stacked sMRI feature guided by the EEG feature. Finally, this work reshapes $C_{a}$ and $C_{b}$ into $R^{\left(G\times H\times W\right)}$ and performs feature concatenation on $C_{a}$ with A, and $C_{b}$ with B to obtain the final stacked features. It can be calculated by Equations 10, 11

$$A_{\mathrm{refined}}=N\text{©}C_{a},$$ (10)

$$B_{\mathrm{refined}}=M\text{©}C_{b},$$ (11)

where $A_{\mathrm{refined}}\epsilon R^{\left(G\times H\times W\right)}$ and $B_{\mathrm{refined}}\epsilon R^{\left(G\times H\times W\right)}$ represents the final EEG features and sMRI features, while N and M represent the initial input EEG features and sMRI features, respectively.

### 3.6 Multimodal model structure

**FIGURE 4:**
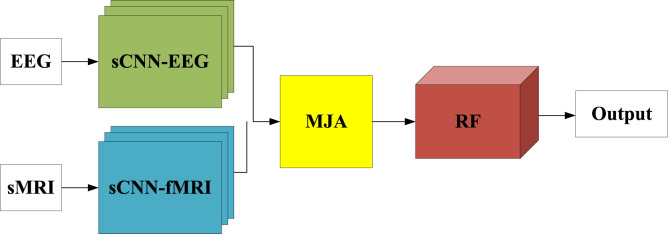
Schematic diagram of MCNNRF module.

The final framework of this work is a combination of sCNN-EEG, sCNN-sMRI models, and fused modality models. The sCNN-EEG and sCNN-sMRI models are responsible for extracting features from the corresponding modalities. The MAJ module is used to solve the interconnection and matching between multimodal features and to fuse multimodal features. MCNNRF is shown in Figure 4. It is divided into three phases. The first stage is to extract features from the corresponding modalities using single modality models (i.e. sCNN-EEG and sCNN-sMRI). The second phase aims to address the lack of interconnectivity and fusion in the multimodal information extraction process. The features extracted from the two single models are used as the input source of the MAJ module. The third stage is the stacked features formed after fusion and the stacked RF is used for classification.

## 4 Experiments

In this section, the experimental environment and dataset are presented in Section A. Unimodal feature extraction model comparison is provided in Section B. Ablation work is provided in Section C. A Comparison between unimodal and multimodal model is provided in Section D. robustness analysis is provided in SectionE. Finally, Comparison with existing researches is provided in Section F.

### 4.1 Experimental environment and dataset

This work is implemented by using TensorFlow library on an NVIDIA RTX A6000 GPU. The dataset used in this work is provided by the research of Colloby et al. (2016). The dataset contains electroencephalogram (EEG) data from 99 Alzheimer’s disease (AD) patients. However, due to the lack of data in 5 cases, magnetic resonance imaging (MRI) scan images are only available for 89 patients. Among the available cases, there are 45 females with an average age of 75.8 ± 7.3 years. The EEG data of CNHCs (Healthy ControlsCognitively Normal) used is from a public static EEG dataset for epilepsy, and the MRI data is from the public ADNI dataset. Despite the data set being small, the model is relatively intricate, and this often culminates in overfitting of the model. Therefore, the experiments in this work use 10-fold cross-validation to deal with these problems. At the same time, the data set will be divided into 8:2 corresponding to the training set and the test set, where the training set is used for training the model, and the test set is used for testing and evaluating the model. The receiver operating characteristic curve (ROC) Tharwat (2018) is used as the main metric for hyperparameter tuning and finding the best model. This work also evaluated some secondary indicators such as sensitivity (Sn), specificity (Sp), accuracy (Acc), precision (Pre), and Matthew correlation coefficient (Mcc). These indicators can be calculated by Equations 12–16

$$S_{n}=tp/\left(tp+fn\right),$$ (12)

$$S_{p}=tn/\left(tn+fp\right),$$ (13)

$$P_{r}e=tp/\left(tp+fp\right),$$ (14)

$$A_{c}c=\left(tp+tn\right)/\left(tp+tn+fp+fn\right),$$ (15)

$$M_{cc}=\frac{tp\times tn-fp\times fn}{\sqrt{\left(tp+fn\right)\times \left(tp+fp\right)\times \left(tn+fn\right)\times \left(tn+fp\right)}},$$ (16)

where tp is true positive, tn is true negative, fp is false positive and fn represents false negative values. They are calculated from the confusion matrix of the predicted results.

### 4.2 Unimodal feature extraction model comparison

**FIGURE 5:**
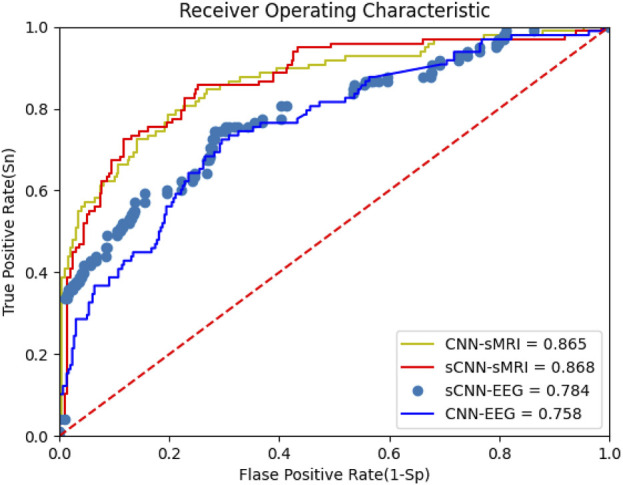
OC of sCNN-EEG, sCNN-sMRI, CNN-EEG and CNN-sMRI.

This work focuses on the feature extraction of EEG and sMRI datausing sCNN-EEG and sCNN-sMRI models, respectively. Prior to determining the sCNN-EEG and sCNN-sMRI models, this work designed some feature extraction model strategies for two modalities, named CNN-EEG and CNN-sMRI respectively. Compared with sCNN-EEG and sCNN-sMRI, CNN-EEG and CNN-sMRI only lacks different multiple pooling layer modules. In this section, the performance of different networks (CNN-EEG, sCNN-EEG, CNN-sMRI, and sCNN-sMRI) is compared. The Receiver Operating Characteristic (ROC) is shown in Figure 5, and the Area Under the Curve (AUC) is calculated. The AUC of sCNN-sMRI is 0.33% higher than that of CNN-sMRI, while the AUC of sCNN-EEG is 2.63% higher than that of CNN-EEG. Other performance indicators are shown in Table 1. Compared with CNN-sMRI, the precision sCNN-sMRI improves to 75.97%, an Mcc improves to 51.35%, and a relatively flat sensitivity. Compared with CNN-EEG, sCNN-EEG has a relatively flat accuracy and a sensitivity improvement of 18.76%. Compared with CNN-EEG/CNN-sMRI, sCNN-EEG, and sCNN-sMRI not only have more multi-branch different convolutional kernels for feature extraction but also strengthen the learning of weak features and use multi-pooling modules to extract deep-level features. At the same time, using stacked connections in the connection allows the extracted features to perform stacked features, which can better integrate hidden features into it.

**TABLE 1:** sCNN-EEG, sCNN-sMRI, various performance indicators of CNN-EEG and CNN-sMRI.

Model	Accuracy (%)	Precision (%)	Sensitivity (%)	Mcc (%)
CNN-sMRI	86.51	71.35	34.07	44.72
sCNN-sMRI	86.84	75.97	36.79	51.35
CNN-EEG	75.80	61.57	22.91	29.37
sCNN-EEG	78.43	61.04	41.67	29.57

### 4.3 Ablation study

**FIGURE 6:**
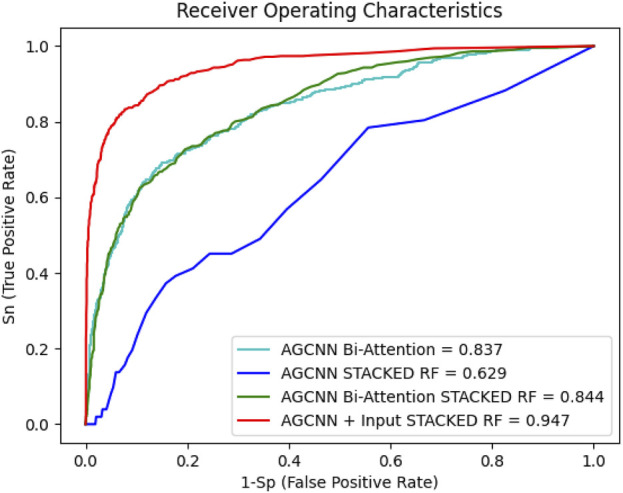
Multimodal strategy ROC

After analyzing the various performance indicators of the centralized single-mode feature extractor, a model for the feature extractor is selected. The feature extractor is used to extract multimodal features, which are then stacked together. The multimodal features are fused and classified using different strategies. Initially, a simple concatenation matrix method is used to fuse the multimodal features, and the performance of the model is evaluated. However, it is observed that simple splicing methods does not directly improve and enhance the performance and classification ability of multimodal models. For this purpose, this work has designed several strategies for multimodal fusion. For example, using a bimodal attention mechanism for fusion. The multimodal model of EEG and sMRI can solve the correlation and cooperation between the modalities and can also classify AD well. Table 2 shows the performance indicators of each strategy, and their ROC curves are shown in Figure 6.

**TABLE 2:** Performance indicators of multimodal models.

Model	Accuracy (%)	Precision (%)	Sensitivity (%)	Mcc (%)
MCNNBA	83.73	73.68	41.67	40.14
MCNNcRF	62.93	51.20	51.09	37.64
MCNNBARF	84.43	81.88	72.36	43.10
MCNNRF	94.75	85.12	80.88	75.34

Figure 6 shows the performance curve of different multimodality models. Even with RF as the classifier, MCNNcRF accuracy is only 62.93%. MCNNBA uses dual attention fusion for multiple modalities, with an accuracy of 83.73%. Compared with MCNNcRF, the accuracy of MCNNBA is much higher than that of MCNNcRF. The main reason is that MCNNBA’s dual attention fusion module is more focused on the connections between multimodals. When MCNN uses Bi-Attention and adds RF for classification, the accuracy rate is 84.43%, because RF enhances its ability to classify stacked features. Finally, when MCNNBA adds RF, the accuracy rate reaches 94.75%. Compared with the previous strategies, this model uses the fusion module to cooperate and deeply fuse the features after extracting the two modal features. Table 2 shows the MCNNRF strategy outperformed all other strategy models in this experiment, demonstrating superior performance in terms of accuracy, precision, sensitivity and Mcc values. When compared with MCNNcRF, the accuracy of MCNNRF is elevated by 31.82%, precision is amplified by 11.44%, and sensitivity is increased by 39.21%. As shown in Table 2 and Figure 6, among different multimodal fusion strategies, MCNNRF with the MJA module shows the best performance in accuracy, precision, sensitivity and Mcc values. Although both MCNNBA and MCNNBARF use bimodal attention mechanisms for fusion, MCNNBARF outperforms MCNNBA in all performance metrics due to its utilization of RF to enhance classification capabilities. Nevertheless, MCNNBARF still falls short of MCNNRF’s performance, as the bimodal attention mechanism only enhances feature extraction capabilities without exploring and amplifying the intercommunication and complementarity between modalities.

### 4.4 Comparison between unimodal and multimodal model

EEG modalities and sMRI modalities are fed into the model by the multimodal model as output sources. Compared to the unimodal model, the multimodal model diversifies the input. At the same time, the multimodal model fuses the characteristic enhancement features between the different modalities, and obtains a higher accuracy. Their various performance indicators are shown in Table 3. It is obvious that the multimodal model is the optimal model, and the accuracy is increased by 8.91% and 16.32% compared with the sCNN-sMRI and the sCNN-EEGl, respectively. At the same time, other parameters have been greatly improved. Overall in this experiment, MCNNRF is far superior to the unimodal model.

**TABLE 3:** Performance indicators of multimodal models.

Model	Accuracy (%)	Precision (%)	Sensitivity (%)	Mcc (%)
sCNN-sMRI	86.84	75.97	36.79	51.35
sCNN-EEG	78.43	61.04	41.67	29.57
MCNNRF	94.75	85.12	80.88	75.34

### 4.5 Robustness analysis

**FIGURE 7:**
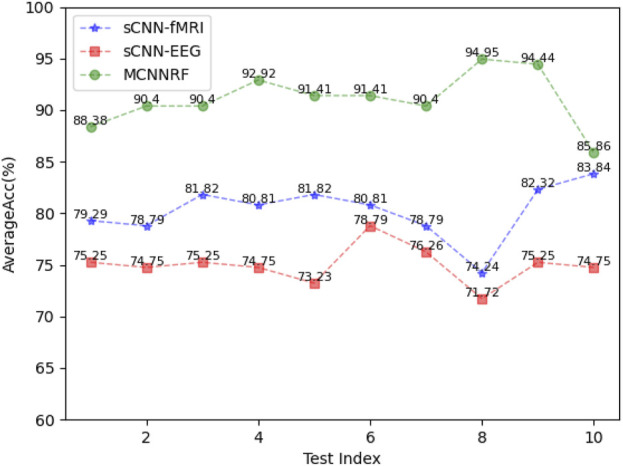
Performance indicators of sCNN-sMRI, sCNN-EEG, and MCNNRF.

To analyze the robustness of the proposed models, 10 independent experiments are performed on each model. The experimental results, measured in terms of accuracy, are presented in Figure 7 The MCNNRF model exhibits obvious advantages, outperforming both unimodal models (sCNN-EEG and sCNN-sMRI) consistently. Even the worst-performing MCNN model surpasses the best-performing sCNN-EEG and sCNN-sMRI models by 7.07% and 2.02%, respectively. Additionally, the curves of all three models demonstrate stability and consistency throughout the experiments.

### 4.6 Comparison with existing researches

#### 4.6.1 Compared to unimodal

In this section, the performance of MCNNRF is compared with advanced AD unimodal diagnostic models. The comparison results are shown in Table 4. The accuracy performance comparison of each model shows that the MCNNRF is the best performing architecture with the highest accuracy. However, when considering the single EEG mode, the accuracy does not show significant differences. Although MCNNRF achieves approximately 1.75% higher accuracy than the other models, there is still a gap compared to DPCNN in terms of accuracy. The reason for this is that their dataset is relatively small and they chose to build their model using DPCNN, which is more suitable for one-dimensional data. The purpose is to increase the convolutional kernel to enhance the learning ability of one-dimensional data and prevent overfitting and gradient explosion issues. LMCN achieves an accuracy of 98%, reaching high levels of precision and sensitivity. The MCNNRF model achieves an accuracy of only 94.75%. This discrepancy primarily stems from LMCN’s application of bidirectional long short-term memory networks to analyze time series predicated on EEG characteristics. Concurrently, LMCN leverages CNN to probe into the relationship between different channels and brain signals. The fusion of these techniques fully harnesses the characteristics of EEG, leading to high accuracy. Adazd-Net achieves an accuracy of 98.51%, a precision of 97.29%, and a sensitivity of 1. This remarkable performance is attributed to Adazd-Net using an interpretable boosting machine as a predictor and employing a designed adaptive and flexible Analytic Dyadic Zernike (ADZ) wavelet transformation for processing EEG data. The adaptive and flexible ADZ wavelet transformation automatically adapts to EEG variations and identifies the most discriminative channels. Compared to LMCN and Adazd-Net, this work significantly differs in EEG data processing. They focus more on the impact of the relationship between channels and EEG on AD, while this work emphasizes the relationships between multiple modalities and does not delve into a detailed analysis of EEG channels. The accuracy of MCNNRF is 6.05% and 4.35% higher than Fuzzy-VGG and VGG16 respectively. However, when compared to Fuzzy-VGG, the accuracy and sensitivity of this work are still lag slightly behind. The reason is that Fuzzy-VGG uses fuzzy C-means to modify image pixels for MRI to achieve the effect of implicitly marking the lesion area. At the same time, the Fuzzy-VGG adopts stacked small kernel convolution, which can obtain more useful information in complex images in a given area. Although the method of Fuzzy-VGG achieves a better result, it cannot directly detect the given area and reduce the interference of useless information. MCNNRF is 3.35% more accurate compared to VGG16. VGG16 uses a large data set. However, the data set of MCNNRF is small, and the feature extraction ability of the model has not been enhanced, so there are not enough features for learning classification. To compared with MRN, MCNNRF is 2.89% less accurate and 2.45% less sensitive. The reason for this significant gap is that MRN uses multi relational inference networks to learn MRI through spatial information correlation and topology. Therefore, MRN is possible to obtain multiple types of inter-regional relationships. MCNNRF extracts deep features from MRI data. The accuracy of IDA-Net is 2.05% lower than that of MCNNRF, but the sensitivity is 11.02% higher. IDA-Net uses the Transformer structure to classify AD, and the dataset used in this method is relatively large.

**TABLE 4:** Performance indicators of multimodal models.

Model	Modal type	Accuracy (%)	Precision (%)	Sensitivity (%)	Mcc (%)
DPCNN Fouad and Labib (2023)	EEG	93.0	95.8	-	-
LMCN Imani (2023)	EEG	98	1.00	97	-
Adazd-Net Khare and Acharya (2023)	EEG	98.51	97.29	100	-
Fuzzy-VGG Yao et al. (2023b)	MRI	88.7	92.9	91.7	72.5
VGG16 Sharma et al. (2022)	MRI	90.4	90.5	-	-
MRN Zhang et al. (2023b)	MRI	97.64	-	83.33	-
IDA-Net Zhao et al. (2023)	MRI	92.7	-	91.9	-
MCNNRF	EEG + sMRI	94.75	85.12	80.88	75.34

### 4.7 Compared to multimodal

This work is the first work to explore the application of deep learning in combination with EEG multimodality for AD diagnosis. Therefore, this work compares with most advanced multimodal methods. The performance comparison between the different models is shown in Table 5. Compared to CNN + ANN model, MCNNRF shows relatively lower accuracy and sensitivity. The reason is that CNN + ANN has conducted deep mining of clinical and biological information. Firstly, CNN is used to extract features from images, and then a fusion module is designed using ANN to fuse and classify features. MCNNRF model lacks the supplementation of auxiliary information like clinical data and does not utilize feature transformation techniques to reduce feature dimensionality differences. Compared to HMGD, MCNNRF shows relatively lower accuracy and sensitivity. The specific reason is that HMGD employs graph diffusion methods to enhance the representation capability of multimodal data, thereby strengthening the measurement of multimodal similarity. However, MCNNRF is more focused on cross-modal collaboration and correlation. In the comparison on Accuracy, the performance of MCNNRF is on par with OLFG. The difference between MCNNRF and OLFG lies in one utilizing a multimodal combination of EEG and MRI, while the other employs MRI and PET. OLFG focuses more on the variations of various information in brain images. MCNNRF considers changes in brain image information, while also focusing on information differences that occur over time. In summary, this work explores the application of multimodal EEG in AD. Compared to MCNNRF, the accuracy of 3D-CNN-BRNN increases 1.25% and the sensitivity value increases 11.12%. The main reason is that the 3D-CNN-BRNN dataset owns a clear time series, with mobile MRI data spanning 6 months. At the same time, bidirectional recurrent neural networks are used to recognize the time series. 3DCNN is used to extract MRI features, and then AD is classified by auxiliary information. However, MCNNRF has the different time span as this method for controlling datasets, and there is also no corresponding time series for recognition. Compared to MCNNRF, MCAD has a 0.68% lower accuracy, and MCAD uses MRI and PET as well as some auxiliary modal information. MCAD uses a cross attention mechanism to fuse modalities, while MCNNRF performs deep feature mining on modalities and finally performs fusion.

**TABLE 5:** Comparison between MCNNRF and multimodal models.

Model	Modal type	Accuracy (%)	Precision (%)	Sensitivity (%)	Mcc (%)
CNN + ANN Wang et al. (2023a)	MRI + profile ect	96.2	97.4	-	
HMGD Wang et al. (2023b)	PET + gene	96.4	97.8	-	
OLSL Chen et al. (2023)	MRI + PET	94.7	89.0	-	
3D-CNN-BRNN Pang et al. (2021)	MRI + dc + cs	96.00	92.00	-	
MCAD Zhang et al. (2023a)	sMRI + PET + CSF	94.07	-	-	
MCNNRF	EEG + sMRI	94.75	85.12	80.88	75.34

## 5 Discussion

The high accuracy of the MCNNRF model can be attributed to the effective cooperation between sCNN - EEG and sCNN - sMRI in feature extraction. The MJA module plays a crucial role in enhancing the correlation between modalities, enabling the model to capture more comprehensive information related to AD. For example, the EEG data provides high - temporal - resolution information, while the sMRI data reflects the structural changes of the brain. The MJA module effectively combines these two types of information, leading to improved diagnostic performance. However, the MCNNRF model also has some limitations. The relatively small dataset used in this study may limit the generalization ability of the model. Additionally, the model only considers EEG and sMRI data, ignoring other potentially important information such as patient history and genetic factors. Future research could focus on expanding the dataset and incorporating more modalities to improve the model’s performance. Previous studies mostly used single - modality data or independent training of multimodal models, lacking the exploration of the correlation between modalities. In contrast, our MCNNRF model uses the MJA module to promote the collaboration between EEG and sMRI modalities. Compared with the study in Colloby et al. (2016) that uses manual feature extraction, our model automatically extracts features through deep learning, reducing subjective bias. And compared with Jesus et al. (2021), our model shows better performance in multimodal fusion and classification. This study is the first to explore the combination of deep learning and EEG multimodality for AD diagnosis. The proposed MCNNRF model provides a new approach for AD diagnosis, which has potential application value in clinical practice. The model’s high - performance multimodal fusion and classification ability can help doctors make more accurate AD diagnoses, contributing to the early detection and treatment of AD.

## 6 Conclusion

In conclusion, this work presents a multimodal AD diagnostic model integrating EEG and sMRI data. It designs sCNN-EEG and sCNN-sMRI for feature extraction, and the classification performance is improved by incorporating RF into the classifier. Comparative experimental results also demonstrate that the proposed diagnostic model is competitive with the state-of-the-art methods for multimodality-based AD diagnosis. Simultaneously, this work pioneers the exploration of deep learning amalgamated with EEG multimodality in the realm of AD diagnosis. It holds promising potential to serve as a viable option for Alzheimer’s Disease diagnosis in the forthcoming future. The results show that MCNNRF achieves state-of-the-art overall performance compared to existing multimodal AD diagnostic models. Furthermore, the results of the ablation experiment demonstrate the effectiveness of the MJA block and deep introduction of RF. It is important to acknowledge that this work has two limitations. On the one hand, MCNNRF only takes EEG and sMRI as input, while ignoring the patterns of patient history. On the other hand, MCNNRF can only handle complete multimodal data and is not suitable for the absence of a certain modality. Therefore, future work will focus on introducing patient history into the proposed framework and adjusting the model structure to handle missing patterns.

## 7 Futuer Works

The multimodal joint attention mechanism (MJA fusion module) proposed in this study provides an effective framework for combining electroencephalogram (EEG) and structural magnetic resonance imaging (sMRI) data with significant improvements in feature extraction and fusion. Future studies can further explore more complex multimodal data fusion strategies, such as the introduction of functional magnetic resonance imaging (fMRI) and near-infrared spectroscopy (NIRS), and the application of deep learning techniques, such as self-attention mechanisms and graph neural networks, to improve the expressiveness and robustness of multimodal fusion and enhance the accuracy of clinical diagnosis. For Alzheimer’s disease (AD), the MJA module can be extended to be applied to early diagnosis and prediction of AD, combining EEG and sMRI data to more comprehensively assess EEG activity and structural changes, constructing a multimodal early diagnostic system, and realizing dynamic tracking of AD patients and evaluation of treatment effects. The potential of EEG as a biomarker for AD should be further explored to provide data support for personalized prediction models. In addition, future research should also focus on the personalization of the model, customizing the fusion model based on the patient’s age, gender, and genetic background, as well as improving the interpretability and clinical applicability of the model to achieve real-time, automated AD detection and integration with healthcare information systems to provide adjunctive diagnostic support.

## Data Availability

The original contributions presented in the study are included in the article/supplementary material, further inquiries can be directed to the corresponding authors.
